# Prediction models for intraventricular hemorrhage in very preterm infants: a systematic review

**DOI:** 10.3389/fped.2025.1605145

**Published:** 2025-06-04

**Authors:** Ping Xiong, Yonggang Wei, Lei Li, Houxin Kang, Zhangbin Yu, Hong Tang, Yuanlin Pu

**Affiliations:** ^1^ Department of Neonatology, The Central Hospital of Enshi Tujia and Miao Autonomous Prefecture, Enshi, Hubei, China; ^2^ Department of Neonatology, Shenzhen People’s Hospital (the Second Clinical Medical College, Jinan University; The First Affiliated Hospital, Southern University of Science and Technology), Shenzhen, Guangdong, China; ^3^ Division of Neonatology, Shenzhen Yantian District People’s Hospital, Shenzhen, Guangdong, China

**Keywords:** intraventricular hemorrhage, very preterm infants, prediction, model, systematic review

## Abstract

**Objective:**

To provide an overview and critical appraisal of prediction models for Intraventricular hemorrhage (IVH) in very preterm infants.

**Methods:**

Our comprehensive literature search encompassed PubMed (MEDLINE), Embase, Web of Science, the Cochrane Library along with targeted searches of the Chinese Medical Association's online journal platform (up to 8 February 2025). We examined relevant citations during full-text review and thoroughly evaluated them for inclusion. We included studies that reported the development and/or validation of predictive models for IVH in preterm infants born at <32 weeks. We extracted the data independently based on the TRIPOD-SRMA checklist. We checked for risk of bias and applicability independently using the Prediction model Risk Of Bias Assessment.

**Results:**

A total of 30 prediction models from 11 studies reporting on model development and 2 models from 2 studies reporting on external validation were included in the analysis. The most frequently reported outcome in both model development studies (54.5%) and model validation studies (50%) was IVH I-IV. The most frequently used predictors in the models were gestational age (43.33%), followed by sex (36.67%), antenatal corticosteroids (33.33%), diastolic blood pressure (33.33%), birth weight (30%), and mean airway pressure (30%). The median C-statistic reported at model development was 0.83 (range 0.74–0.99). The majority of the included studies had a high risk of bias, mainly due to suboptimal analysis and mishandling of missing data. Furthermore, small sample sizes and insufficient numbers of event patients were observed in both types of studies. No meta-analysis was performed because no two studies validated the same model in comparable populations. We summarized performance metrics (e.g., C-statistic) descriptively.

**Conclusion:**

The included studies may still be flawed to a certain extent. It is recommended that future studies augment the sample size and number of events, whilst ensuring that any missing data is addressed in a rational manner. Furthermore, the statistical analysis should be optimised, and the study made transparent for the purpose of model generalisation.

## Introduction

Globally, between 2010 and 2020, approximately 15% of all preterm births occurred at less than 32 weeks of gestation (1). Intraventricular hemorrhage (IVH) was common in premature newborns (2). IVH of prematurity occurs in 20%–38% of infants born <28 weeks gestational age and 15% of infants born in 28–32 weeks gestational age (3). A systematic review and meta-analysis found that the overall prevalence of IVH in preterm infants has not changed significantly since 2007 (4).

IVH can lead to ventricular dilatation, hydrocephalus, and other associated complications (5, 6). Long-term follow-up studies have shown that IVH can lead to cerebral palsy, hydrocephalus-related sequelae, epilepsy, deafness, blindness, and autism spectrum disorders (7–10).

In recent years, a number of models have been developed to predict the probabilistic risk of neonatal IVH to support clinical decision-making. However, these models incorporate diverse variables and vary in quality, causing confusion among clinicians regarding which model to adopt or recommend. Moreover, no systematic review has been published to date that comprehensively evaluates predictive models for IVH in very preterm infants based on national and international data.

The purpose of this study was to evaluate the development, validation, and clinical application of a prediction model for the occurrence of intraventricular hemorrhage (IVH) in very preterm infants during their hospitalization in the NICU, thereby providing a reference for clinical practice and future research.

## Methods

This systematic review of all studies on prediction models for IVH in very preterm infants is reported according to Transparent reporting of multivariable prediction models for individual prognosis or diagnosis: checklist for systematic reviews and meta-analyses (TRIPOD-SRMA) (11). Details of the protocol for this systematic review were registered on PROSPERO (ID: CRD42025649529).

### Search strategy

PubMed (MEDLINE), Embase, Web of Science and the Cochrane Library were systematically searched from inception through to 8 February 2025, for studies reporting prediction models of IVH in very preterm infants. We identified relevant studies and maximized search accuracy using the following terms: intraventricular hemorrhage, preterm infants, and prediction. To prevent omission of potentially eligible studies, we examined relevant citations during full-text review and thoroughly evaluated them for inclusion. Additionally, considering the authoritative status of Chinese Medical Association journals in China, we conducted a targeted search on the Chinese Medical Association website using the terms “intraventricular hemorrhage AND prediction model”. To comprehensively include all relevant data in the regression analysis, we conducted a targeted full-text screening. Supplementary Material S1 shows the search strategies. The search was not limited by language.

### Eligibility criteria

The following criteria must be met by the included studies: (1) The target population was preterm infants born at <32 weeks; (2) the study detailed prediction model development and/or external validation; (3) the primary prediction outcome was IVH, defined on standard head ultrasound; (4) the classification system developed by Papile (12) was utilised to categorise the severity of IVH (Grade I-subependymal hemorrhage, Grade II-intraventricular hemorrhage without ventric- uIar dilatation, Grade III-intraventricular hemorrhage with ventricular dilatation, Grade IV-intraventricular hemorrhage with parenehymal hemorrhage); (5) the model was constructed with at least two predictor; (6) the purpose of the model was for predicting IVH in preterm infants from birth. There was no yearly limit on included studies. Articles were ineligible if the outcome to be predicted was the composite outcome “IVH and/or death” or “IVH and/or other”; if the study did not have a prediction model and/or an externally validated study; or if the article was a conference abstract, review, or letter.

### Study selection and data extraction

Two reviewers independently screened the titles, abstracts, and full texts. In case of discrepancies, a third reviewer was involved to establish consensus. The reviewers used a standardized data extraction form based on the TRIPOD-SRMA checklist (11). The following items were extracted from the studies on prediction model development: author, year of publication, country, study design, study population, predicted outcome, intended moment of model use, number of models, number of candidate predictors, predictors included in the final model, sample size, number of events, missing data approach, variables election method, modeling method, assessment of model performance, model presentation, and internal validation method. The following items were extracted from the prediction model external validation studies: author, year of publication, country, study design, study population, predicted outcome, intended moment of model use, sample size, number of events, missing data approach, and assessment of model performance. The events per variable (EPV) was defined as the number of events divided by the number of candidate predictor variables used (13).

### Assessment of bias

We assessed risk of bias using the PROBAST tool (14, 15), which is specifically developed for systematic evaluation of prediction models. Unlike generic observational study tools (e.g., ROBINS-I for causal inference or Newcastle-Ottawa Scale for non-prediction cohorts), PROBAST evaluates four critical domains—participant selection, predictor measurement, outcome definition, and analytical methods—while also grading applicability to clinical practice. This dual assessment is essential because prediction models require validation of both methodological rigor and real-world utility, a feature not comprehensively addressed by other tools.

The risk of bias (ROB) and applicability of each article was assessed with PROBAST (14, 15), a tool which consists of 20 signalling questions across four domains (participants, predictors, outcomes, and analysis). The ROB of the original studies was classified as high, low, or unclear for each domain via comprehensive evaluation. It was determined that a study would be classified as overall low ROB only if each domain had low ROB. In order to ascertain the applicability of each item, they were assigned a rating of high, low, or unclear based on the extent to which the review questions corresponded to the study, according to the three dimensions (participants, predictors, and outcomes). This evaluation process was conducted by two researchers; in the event of disagreement, a third researcher was involved in the discussion and made the final decision.

### Model performance

In terms of model performance, discrimination is frequently quantified by the C statistic, which is the most commonly used measure for assessing the discriminative ability of models with binary outcomes. Typically, a C-statistic below 0.6 is considered poor, a C-statistic between 0.6 and 0.75 is considered possibly helpful, and a C-statistic above 0.75 is considered clearly useful (16).

## Results

**Figure 1:**
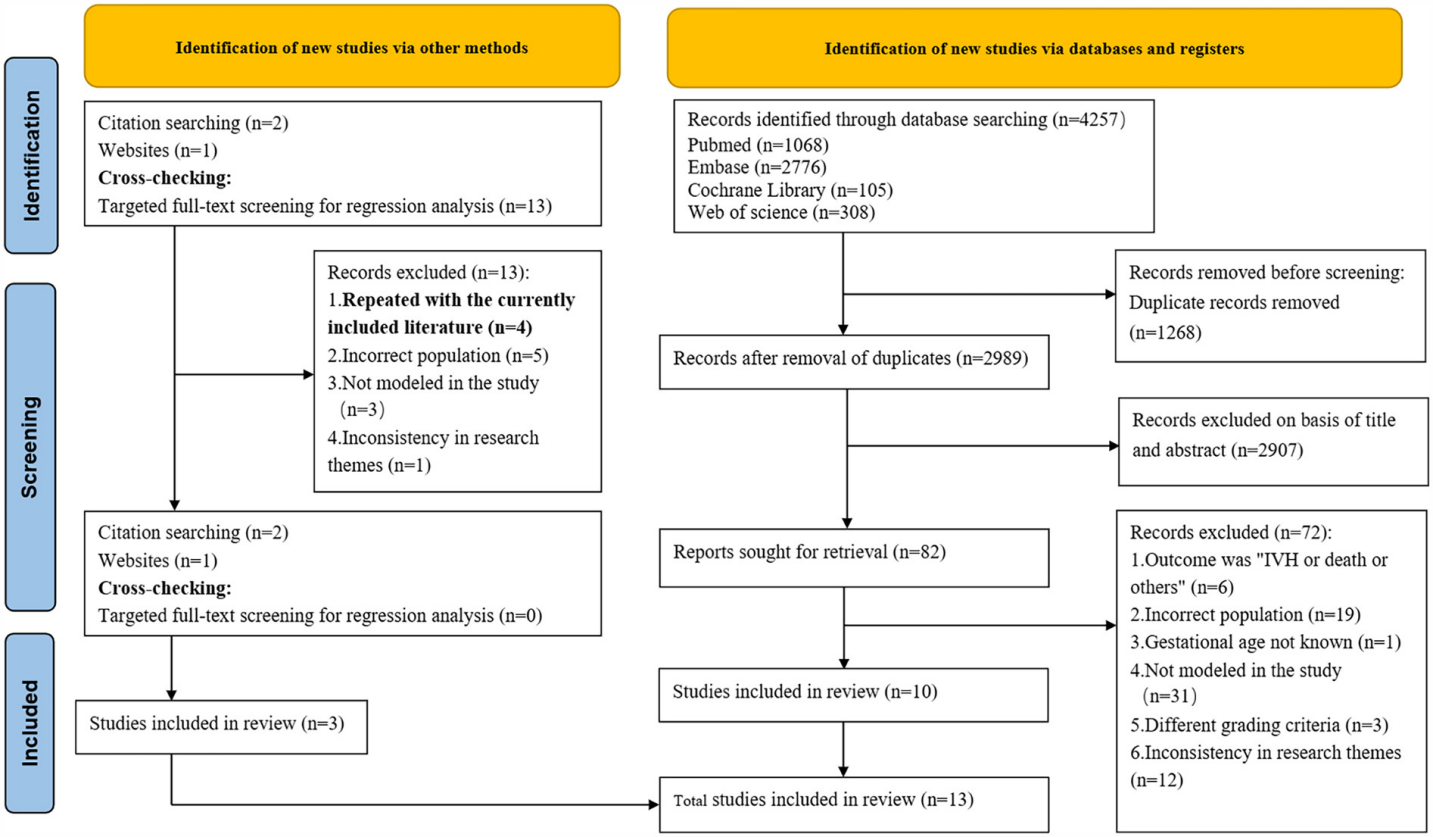
Preferred reporting items for systematic reviews and meta-analyses (PRISMA) flow diagram.

A preliminary investigation was conducted by searching the PubMed, Embase, Cochrane Library and Web of Science databases, which yielded a total of 4,257 articles. After excluding duplicates, 2,989 articles remained, which were then subjected to a thorough review of their titles and abstracts, leading to the selection of 81 articles. Subsequent to a comprehensive review of the full texts, ten articles (17–26) were deemed to meet the inclusion criteria. Additionally, during full-text review, citation tracking identified two additional eligible studies, while one further qualifying study was located through the Chinese Medical Association website search. Additionally, through cross-checking, we identified four primary studies that overlapped with studies already included in our systematic review (18, 19, 22, 27). The final number of studies included in the system was 13 (17–29). The specific search process is illustrated in Figure 1. Eleven studies (17–21, 23, 25–29) described model development without external validation, and two studies (22, 24) described external validation without model updating. (Please refer to the Supplementary Appendix Table S2 for a list of literature that was excluded).

### Characteristics of studies describing IVH prediction model development

A total of eleven studies (17–21, 23, 25–29) were found to have described the development of predictive models for IVH, with a total of 30 models having been developed. The main characteristics of the models in these studies are shown in Table 1, including the study design, the study population, the moment of model use, and the main reasons for the differences that developed between the multiple models in the unified study. Table 2 shows the study and performance characteristics of the developed models.

**Table 1:** Design characteristics of the 11 studies describing the development of IVH prediction models.

Study	Year of publication	Country	Study design	Years of data	Study population (weeks)	Missing data	Intended moment of model use	Model, n	Differences between models caused by differences in the following
Cucerea et al. (19)	2024	Italy	Retrospective case-control	2017–2022	≤28	NR	Within the first 4 days after birth	1	NA
Han et al. (28)	2024	Korea	Retrospective cohort	2013–2022	<32	Zero-padding	Within the first 14 days after birth	5	Modelling method
Sidorenko et al. (18)	2024	Germany	Retrospective case-control	2006–2016	23–30	Delete missing values and extend low-frequency measurements	Within the first 14 days after birth	7	Different predictors
Wang et al. (17)	2024	China	Retrospective case-control	2022–2023	<32	No missing data	Within the first 3 days after birth	1	NA
Ushida et al. (20)	2023	Japan	Registry	2006–2015	<32	No missing data	At birth	6	Modelling method
Liu et al. (29)	2023	China	Retrospective case-control	2017–2021	<32	No missing data	Within the first 14 days after birth	1	NA
Ushida et al. (21)	2021	Japan	Registry	2006–2015	<32	No missing data	At birth	1	NA
He et al. (23)	2019	China	Retrospective cohort	2015–2018	24–32	NR	Within the first 5 days after birth	1	NA
Huvanandana et al. (25)	2017	Australia	Prospective observational	NR	<30	No missing data	Within the first 7 days after birth	5	Different predictors
Heuchan et al. (27)	2002	Australia and New Zealand	Retrospective cohort	1995–1997	<30	Delete missing values	Within the first 10 days after birth	1	NA
Van et al. (26)	1987	Netherlands	Prospective cohort	1983	<32	NR	At birth	1	NA

IVH, intracranial hemorrhage; NA, not applicable; NR, not reported.

**Table 2:** Study and performance characteristics of the developed prediction models.

Study	Outcome	Sample size	Events	EPV	Univariable analysis	Modeling method	Predictoes, *n*	C-statistic	Model presentation	Calibration	Internal validation
Cucerea et al. (19)	IVH I-IV	134	48	4.8	Yes	LR	5	NR	Formula	NR	NR
Han et al. (28)	IVH I-IV	523	48	3.4	No	ET	All 14	0.999	NR	Precision-recall curve	Split-sample validation
						WE		0.997		NR	
						RF		0.999			
						KNN		0.983			
						NN		0.981			
Sidorenko et al. (18)	IVH I-IV	254	136	8	Yes	LR	5	0.84	All scoring system	NR	Time-based Split Validation
							6	0.74			
							4	0.81			
							3	0.79			
							6	0.84			
							4	0.82			
							6	0.85			
Wang et al. (17)	IVH I-IV	241	89	6.8	Yes	LR	4	0.814 (0.762–0.869)	Nomogram	Calibration curve, Decision curve	Split-sample validation
Ushida et al. (20)	IVH III-IV	20,650	1,126	93.8	No	RR	All 12	0.773	NR	NR	Split-sample validation
						FCNNs		0.774			
						SVM		0.772			
						RF		0.765			
						AdaBoost		0.771			
						GBDT		0.771			
Liu et al. (29)	IVH II-IV	512	52	6.5	Yes	LR	5	0.818 (0.757–0.878)	Nomogram	Calibration plots, HL-test	Bootstrap
Ushida et al. (21)	IVH III-IV	20,650	1,126	93.8	Yes	LR	6	0.78 (0.75–0.80)	Formula	Calibration plots, HL-test	Split-sample validation
He et al. (23)	IVH III-IV	516	32	3.2	Yes	LR	6	0.83	Scoring system	HL-test	Time-based Split Validation
Huvanandana et al. (25)	IVH I-IV	27	7	0.5	Yes	LR	All 2	0.921	NR	NR	Leave-One-Out Cross-Validation
								0.843			
								0.843			
								0.864			
								0.871			
Heuchan et al. (27)	IVH III-IV	3,772	252	18	Yes	LR	5	0.77	Scoring system	NR	Time-based Split Validation
Van et al. (26)	IVH I-IV	484	140	11.6	Yes	LR	5	NR	NR	NR	NR

IVH, Intraventricular hemorrhage; NR, not reported; LR, Logistic regression; KNN, K-nearest neighbors; RF, Random forest; ET, Extra Trees; WE, Weighted ensembles; NN, Neural networks; RR, Ridge regression; FCNNs, Fully connected neural networks; SVM, Support vector machine; AdaBoost, Adaptive boosting; GBDT, Gradient boosting decision tree; HL-test, Hosmer–Lemeshow test.

### Study design

A total of 11 original studies (17–21, 23, 25–29) were identified, of which 36.3% were retrospective case-control studies (17–19, 29), 27.3% were retrospective cohort studies (23, 27, 28), 18.2% were prospective observational studies (25, 26) and 18.2% were registry studies (20, 21). All studies were developed on the basis of statistical methods. Nine studies (17–19, 21, 23, 25–27, 29) (81.8%) used logistic regression as a predictive modelling approach and two studies (20, 28) (18.2%) used machine learning as a predictive modelling approach.

### Outcome to be predicted

The findings of this predictive model were all indicative of IVH, with the diagnosis being made on the basis of ultrasonography and the grading criteria referring to the Papile grading scale (12). Of the studies undertaken for the development of the model, six original studies (17, 19, 22, 25, 26, 28) developed predictive models for IVH grades I-IV, one original study (29) developed a predictive model for IVH grades II-IV, and the findings of four original studies (20, 21, 23, 27) developed predictive models for IVH grades III-IV. The expected moment of model use was uncertain, partly within 14 days after birth, and in three of the original studies (20, 21, 26) the moment of model use was after birth. All predictive models were built in the NICU.

### Predictors

All 11 original studies reported the number of candidate predictors for the IVH prediction model, with the number of candidate predictors ranging from 8 to 29 and the number of predictors included in the final model ranging from 2 to 14 (see Supplementary Appendix Table S3).

**Figure 2:**
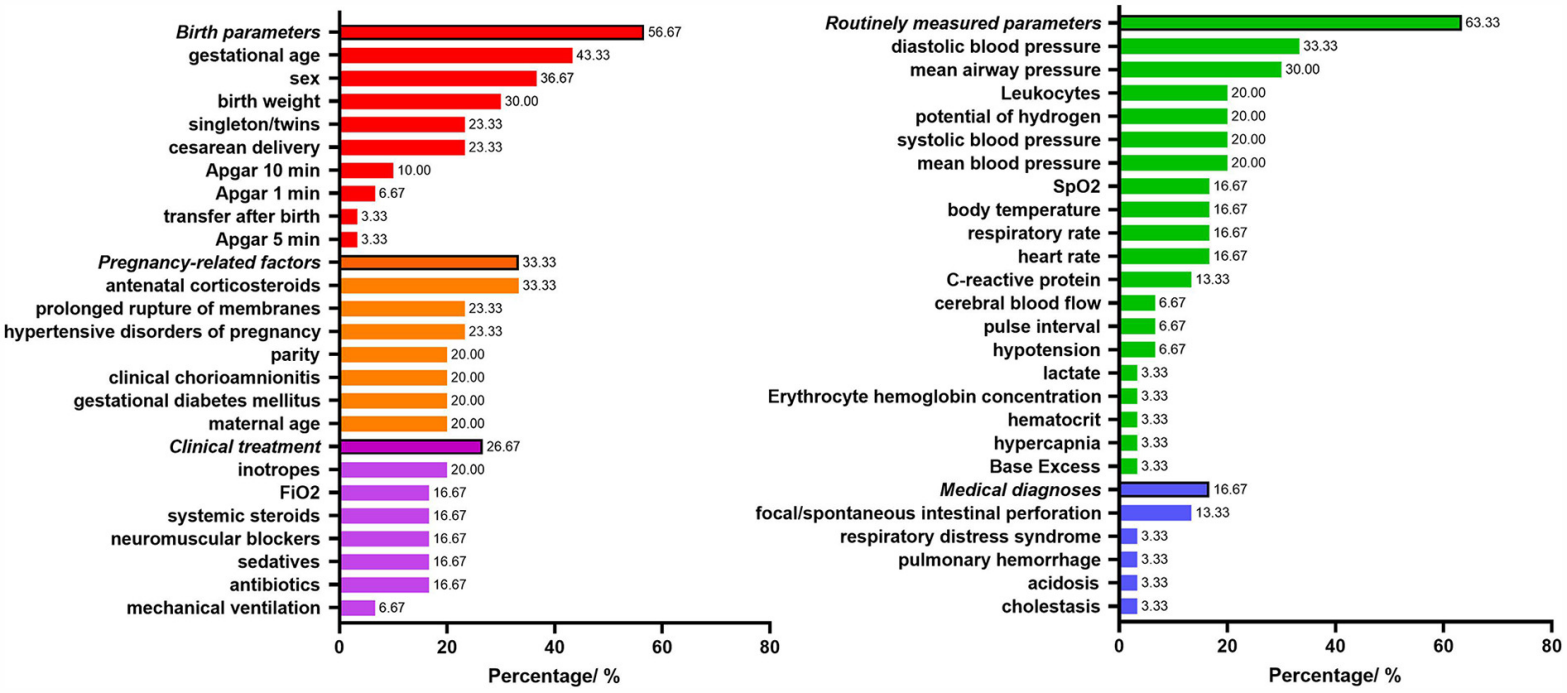
Predictors included in the final development models.

Figure 2 shows the predictors included in the final prediction models. Nineteen models (63.33%) used routinely measured parameters, seventeen models (56.67%) used birth parameters, ten models (33.33%) used pregnancy-related factors, eight models (26.67%) used clinical treatment parameters, and five models (16.67%) related to medical diagnoses parameters. The most frequently included predictor in the 30 prediction models was gestational age (43.33%), followed by sex (36.67%), antenatal corticosteroids (33.33%), diastolic blood pressure (33.33%), birth weight (30%), mean airway pressure (30%).

### Sample size

The development of the models involved a sample size of 27–20,650 participants (median, 512), and a range of 7–1,126 events (median, 89). The EPV ranged from 0.5 to 93.8 (median, 6.8). It was observed that the EPV was less than 10 in 63.6% of the models in which it was calculated.

### Missing data

Of the studies reviewed, 27.3% did not mention missing data, 45.4% had no missing data and 27.3% described methods for dealing with missing data.

### Model presentation

The review encompassed 30 models, which were categorized based on their presentation formats as follows: the majority (56.6%, *n* = 17) lacked specification of presentation format. A substantial proportion (30%, *n* = 9) utilized scoring systems. While 6.7% (*n* = 2) employed formulaic representations and an equivalent percentage (6.7%, *n* = 2) were presented through nomograms.

### Apparent predictive performance

In the context of model development, a total of nine studies (17, 18, 20, 21, 23, 25, 27–29) (81.8%) employed the C-statistic to assess model performance, with values ranging from 0.74 to 0.99 (median 0.83). A total of five models were assessed for calibration. Three models used the Hosmer-Lemeshow test, two used calibration plots, and one used both calibration curve and decision curve. Finally, one study (28) used a precision-recall curve for the optimal model generated by Extra Trees Classifier.

### Internal validation

Of the eleven studies (17–21, 23, 25–29) on model development, only two studues (19, 26) did not perform internal validation, while the remaining nine did (81.8%). The four studies (17, 20, 21, 28) that employed split-sample validations, the three (18, 23, 27) that used time-based split validations, and the one (25) that employed leave-one-out cross-validation, as well as the one (29) that employed bootstrap.

### Risk of bias and applicability

**Figure 3:**
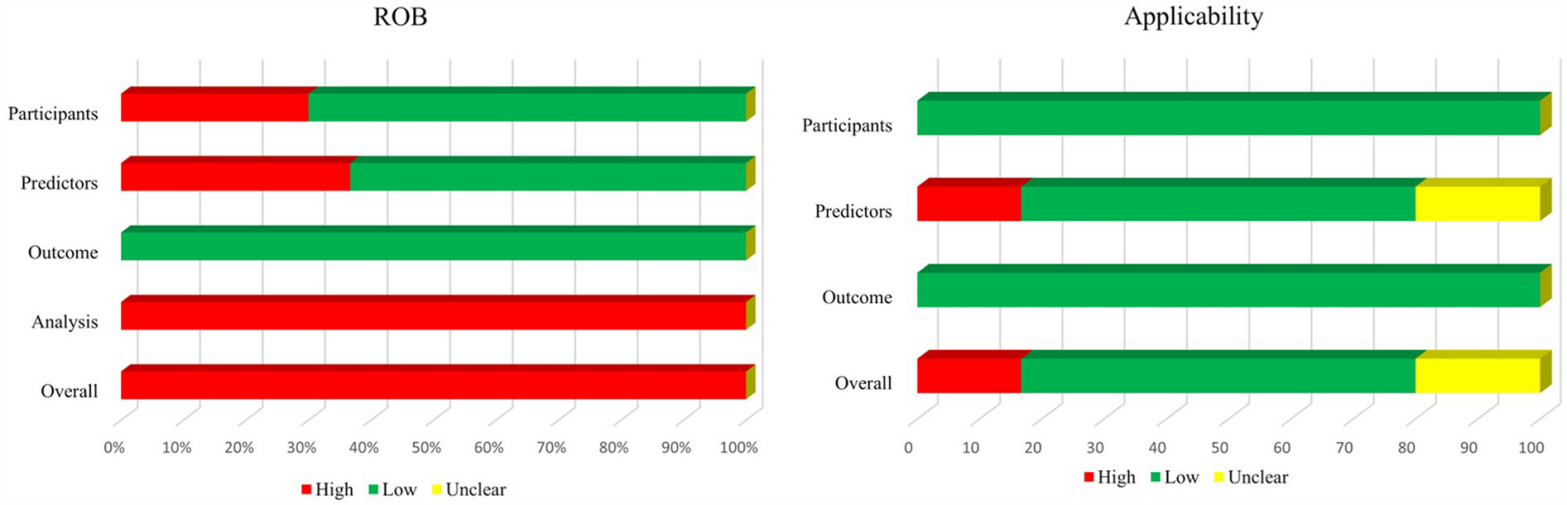
Risk of bias and applicability assessment of developed models using prediction model risk of bias assessment tool (PROBAST).

Figure 3 shows a summary of the ROB and applicability for all developed models. Across all models, there was high participants' domain-related ROB in 30% of the models. For the domain predictors, 63.33% of the models had low ROB, but high ROB had 36.67%. ROB related to outcome was considered low. By contrast, ROB related to the statistical analysis was high in the all models, mostly because of inappropriate handling of missing data, alongside an insufficiency of outcome EPV. Furthermore, there was no consideration of overfitting, and lack of model performance and calibration assessment. In summary, the overall ROB was high across all models.

In terms of applicability concerns, 16.67% of the models were rated as high concern due to potential lack of association between predictors and outcomes, while 20% were classified as unclear concern because predictors were not clearly defined.

### External validation

Two studies (22, 24) conducted external validation independently for CRIB-II (Clinical Risk Indicator fores-II) and SNAP-II (Score for Neonatal Acute).

Siddappa et al. (22) conducted a study with the objective of validating SNAP-II. This was a validation of neonatal IVH grade III-IV. The study design was retrospectively case-controlled, with a sample size of 101 and 15 events. The study did not make mention of missing data, and the C-statistic was 0.78. The predicted moment of time used for the model was 12 h after birth. The study by Galderisi et al. (24) was a validation of CRIB-II and the outcome of the model validation was neonatal IVH grade I-IV. This was a retrospective case-control study with a sample size of 29, a number of positive events of 6, no missing data, a C-statistic of 0.885, and the time of model use was 1 h after birth. The details of the above two external validations are shown in Table 3.

**Table 3:** Design characteristics of the 2 studies describing the external validation of IVH prediction models.

Study	Year of publication	Country	Study design	Years of date	Study population (weeks)	Outcome	Sample size	Outcome events	C-statistic	Intended moment of model use	Model validated
Siddappa et al. (22)	2021	USA	Retrospective case-control	2008–2013	<29	IVH III-IV	101	15	0.78	12 h after birth	SNAPPE-II
Galderisi et al. (24)	2019	Italy	Retrospective case-control	NR	<32	IVH I-IV	29	6	0.885	1 h after birth	CRIB-II

CRIB-II score, clinical risk index for babies II; SNAPPE-II, Score for Neonatal Acute Physiology– Perinatal Extension II.

In the study by Galderisi et al. (24), a separate model for Continuous Glucose Monitoring Linked to an Artificial Intelligence Risk Index was constructed to predict IVH in neonates. However, the predictor was only the glycaemic index, so it was not recorded in the model development.

Among 2 validated models, none had ≥2 external validations meeting our criteria. Thus, quantitative synthesis was not feasible.

**Figure 4:**
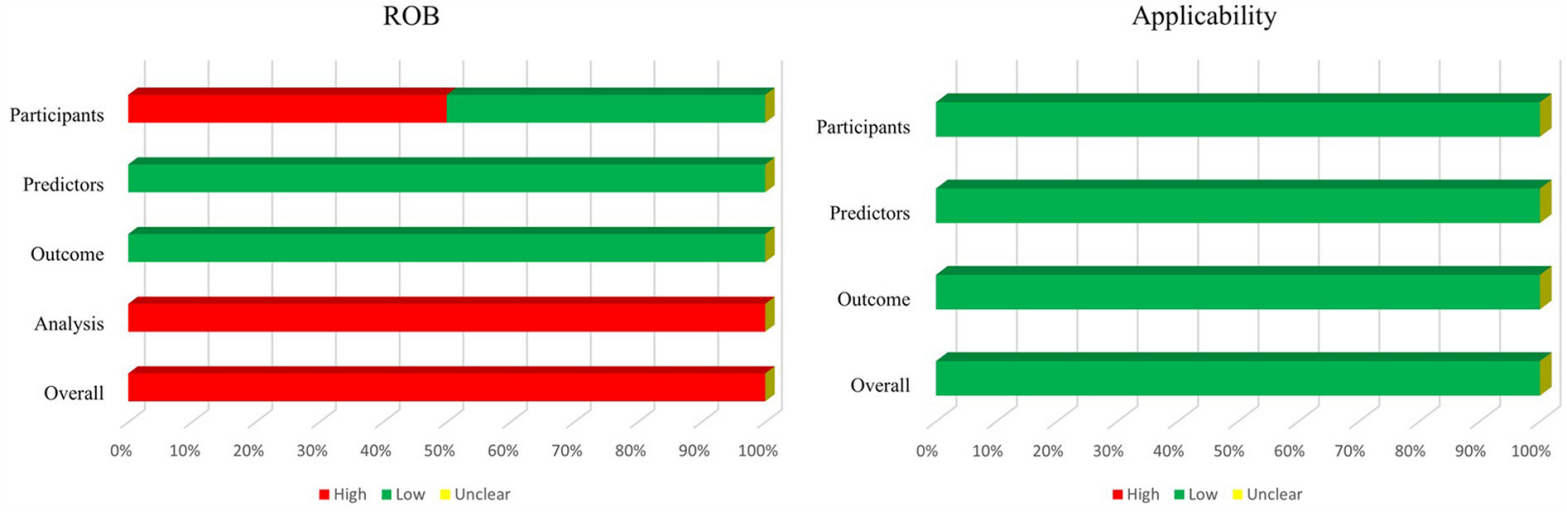
Risk of bias and applicability assessment of externally validated models using prediction model risk of bias assessment tool (PROBAST).

Figure 4 shows a summary of ROB and applicability by domain. Across almost all models, for the domain participants, high and low ROB both had 50%. ROB related to outcome and predictors was low. By contrast, ROB related to the analysis was high in almost all studies, because of an insufficiency of outcome EPV. Both external validation applicability was of low concern.

## Discussion

In this systematic review, we summarize all diagnostic predictive models for the development of IVH in preterm infants born at <32 weeks. The review included 11 studies (17–21, 23, 25–29) focusing on model development and 2 studies (22, 24) describing external validation. High ROB was observed in almost all models, primarily due to inappropriate handling of missing data, insufficient outcome events per variable, lack of consideration of overfitting, and insufficient assessment of model performance and calibration. Additionally, the lack of comprehensive model reporting in several studies hindered external validation and implementation in clinical practice. Conducting a meta-analysis was further hampered by the absence of external validation studies for the same model.

Predictive models are developed to support medical decision making, so it is crucial to clearly define the target population. IVH is a major complication specifically in very preterm infants born before 32 weeks gestation (30), and its incidence has not decreased with changes in medical conditions (4). Long-term follow-up has shown that IVH can lead to adverse outcomes such as blindness, deafness, epilepsy and cerebral palsy (8–10). This review deliberately focused on studies in preterm infants born at <32 weeks, excluding more mature preterm infants.

This study focuses on predicting IVH. The included studies utilized Ultrasonography for IVH diagnosis, which offers the advantages of being cost-effective, easy to use, repeatable, and highly accurate (31). IVH grading was based on the widely accepted Papile classification system (I-IV grades) (12). During model development, some studies (32, 33) employed composite outcomes, such as “IVH and/or death” or “ IVH and/or other.” While these composite outcomes help account for cases where IVH might have occurred had the patient survived (avoiding exclusion bias), not all early deaths would have necessarily developed IVH. Similarly, not all patients with comorbid conditions would have had IVH. Consequently, using models designed for these composite outcomes to predict IVH alone may reduce predictive accuracy. Recently, numerous robust predictive models (34–36) already exist for mortality and other specific conditions, most demonstrating strong performance. In clinical practice, greater accuracy may be achieved by using dedicated models tailored to the specific outcome of interest rather than relying on composite-outcome models.

In these 11 model development studies, GA, sex, DBP, antenatal corticosteroids, MAP, and BW were identified as core predictive parameters, with most data collected within the early postnatal period (≤24 h) (20, 21, 25, 28, 29). However, this narrow timeframe poses a critical limitation: approximately 38% of IVH cases occur after this initial 24-h window (37). Consequently, predictive models relying solely on static early postnatal indicators may fail to capture the dynamic physiological fluctuations integral to IVH pathogenesis. In contemporary neonatal intensive care, continuous hemodynamic monitoring (arterial blood pressure trends) and cerebral oxygenation metrics (via near-infrared spectroscopy, NIRS) are increasingly adopted, providing real-time insights into cerebrovascular autoregulation and metabolic status (38–40). Future predictive models should prioritize the integration of such high-frequency dynamic metrics to better capture the time-sensitive pathophysiology of IVH.

The present systematic review identified 30 distinct prediction models for IVH development, incorporating core parameters and various clinical variables including general measurements, birth parameters, therapeutic diagnostic indicators, and pregnancy-related factors. These models demonstrated variable discriminatory performance, with C-statistic values ranging from 0.74 to 0.99 (90% > 0.75) in development cohorts and 0.78 to 0.885 in external validation sets (see Supplementary Appendix Table S4), indicating generally moderate to good discriminatory ability (16). However, there is a risk of overfitting the model, so there is still a need to have external data for validation and to assess the ability of the model to generalise for the purpose of model generalisation.

Our quality assessment identified methodological limitations in 11 model development studies (17–21, 23, 25–29). Three primary sources of bias were identified: (1) At the study design level, case-control designs in some studies (17–19) potentially introduced selection bias; (2) In predictor selection, certain variables (body temperature) were included without adequate validation of their independent association with IVH; (3) Regarding statistical methodology, multiple issues emerged including variable prescreening through univariable analysis in nine studies (17–19, 21, 23, 25–27, 29) [this may result in the oversight of significant multivariate relationships and an elevated risk of overfitting (41)], insufficient EPV in 81.8% of studies (EPV <20) [In general, studies with EPV lower than 10 are likely to have overfitting, whereas those with EPVs higher than 20 are less likely to have overfitting (15)], and suboptimal internal validation methods [split-sample validation in 63.6% of studies, but it's not a good idea to split the samples, because this can make the model unstable (42)].

In addition, the review highlighted several critical reporting deficiencies that limit clinical applicability and reproducibility, and even affect model credibility. First, performance reporting was incomplete, with 6.6% of studies (19, 26) failing to report discrimination statistics (C-statistic) and 54.5% of studies (18–20, 25–27) omitting calibration analyses. Second, model transparency was compromised in 36.4% of studies (20, 25, 26, 28) due to incomplete model presentation. Third, handling of missing data was inadequate in 27.3% of studies, while another 27.3% failed to report missing data entirely.

External validation studies (22, 24) presented additional limitations, particularly regarding sample size. Both identified validation studies had fewer than 100 outcome events, potentially leading to imprecise performance estimates (43).

Based on the identified limitations, we propose the following methodological refinements for future IVH prediction studies while maintaining necessary flexibility in implementation: Where feasible, prospective multicenter designs should target EPV ≥ 20 to ensure adequate statistical power (15). For validation approaches, bootstrap resampling with optimism correction is strongly recommended over simple data splitting, particularly for smaller sample sizes (41, 42). External validation efforts should ideally incorporate ≥ 100 outcome events from diverse clinical settings to enhance generalizability (15, 43). Most critically, we advocate for complete transparency through comprehensive reporting of all model parameters (including intercept terms), detailed calibration metrics (with graphical plots), and thorough documentation of missing data handling procedures. These reporting standards are essential to enable proper model evaluation, facilitate external validation, and support clinical implementation. When possible, supplementary sharing of analysis code and de-identified datasets through public repositories would further strengthen research reproducibility. Collectively, these evidence-based refinements could substantially improve both the methodological rigor and clinical applicability of future IVH prediction models while accommodating varying research contexts.

To the best of our knowledge, there is currently no systematic evaluation of prediction models for intracranial hemorrhage in extremely preterm infants. This study endeavors to comprehensively review and synthesize the existing relevant prediction models, with the objective of providing a reference for clinical practice and laying the foundation for subsequent research.

This study has several limitations that warrant consideration. First, by excluding infants with gestational age ≥ 32 weeks and studies using composite outcomes, we may have overlooked potentially valuable prediction models from the broader preterm population. More significantly, the field currently lacks any prediction model that has undergone external validation across multiple independent studies - a fundamental requirement for performing meta-analysis of model performance. These limitations underscore important methodological challenges in IVH prediction research and emphasize the need for standardized approaches and coordinated validation efforts in future investigations. In addition, this study excluded literature with inconsistent diagnostic grading criteria, which may have resulted in the inclusion of studies focused on specific clinical scenarios. Although this improves internal logical consistency, the conclusions may not adequately reflect the applicability of the model in settings with widely varying diagnostic criteria, limiting the generalisability of the results.

## Conclusion

This systematic review evaluated 13 studies that developed or validated prediction models for IVH in very preterm infants. Using the checklist for systematic reviews and meta-analyses and PROBAST tool, we identified significant methodological and reporting limitations in the existing literature. To improve future studies, we recommend: (1) ensuring adequate sample sizes for model development and validation, (2) employing multiple imputation to handle missing data appropriately, (3) avoiding reliance on univariable screening for predictor selection, (4) assessing model performance using both discrimination and calibration measures, and (5) applying robust internal validation techniques for newly developed models. These evidence-based recommendations aim to enhance the methodological rigor, transparency, and clinical applicability of future IVH prediction research.
