# Supplementary material for: Prediction models for intraventricular hemorrhage in very preterm infants: a systematic review
Source: Front Pediatr. 2025 Jun 4;13:1605145. doi: 10.3389/fped.2025.1605145 (PMC12174386; doi:10.3389/fped.2025.1605145)
Supplement: Supplementary file 1 [file Table1.docx]

**Supplementary Material 1** Search strategies for PubMed (MEDLINE), Embase, the Cochrane Library and Web of science

| **No.** | **Search terms** | Results |
| --- | --- | --- |
| **Pubmed(MEDLINE)** （2025-2-8） | | |
| #1 | "Cerebral Intraventricular Hemorrhage"[Mesh] | 417 |
| #2 | ((((((((Intraventricular Hemorrhage[Title/Abstract]) OR (Intraventricular Haemorrhages, Cerebral[Title/Abstract])) OR (Intraventricular Haemorrhage, Cerebral[Title/Abstract])) OR (Haemorrhage, Cerebral Intraventricular[Title/Abstract])) OR (Cerebral Intraventricular Haemorrhages[Title/Abstract])) OR (Cerebral Intraventricular Haemorrhage[Title/Abstract])) OR (Intraventricular Hemorrhage, Cerebral[Title/Abstract])) OR (Hemorrhage, Cerebral Intraventricular[Title/Abstract])) OR (Cerebral Intraventricular Hemorrhages[Title/Abstract]) | 9,738 |
| #3 | #1 or #2 | 9,837 |
| #4 | "Infant, Premature"[Mesh] | 68,727 |
| #5 | ((((((((Infants, Premature[Title/Abstract]) OR (Premature Infant[Title/Abstract])) OR (Premature Infants[Title/Abstract])) OR (Preterm Infants[Title/Abstract])) OR (Infant, Preterm[Title/Abstract])) OR (Infants, Preterm[Title/Abstract])) OR (Preterm Infant[Title/Abstract])) OR (Neonatal Prematurity[Title/Abstract])) | 55,380 |
| #6 | #4 or #5 | 88,997 |
| #7 | ((("predict" OR "prediction" OR "predictive" OR "predicted" OR "prognosis" OR "prognostic factor" OR "evaluation" OR "evaluation study" OR "risk factor" OR "risk assessment" OR "regression analysis" OR "logistic model" OR "statistical model" OR "algorithm" OR "multivariate analysis" OR "predictive value of tests" OR "Area Under Curve" OR "Receiver Operator Curve"))) | 5,623,420 |
| #8 | #3 and #6 and #7 | 1,068 |
| **Embase** (2025-2-8) | | |
| #1 | 'brain hemorrhage'/exp | 202,297 |
| #2 | 'cerebral intraventricular hemorrhages':ab,ti OR 'hemorrhage, cerebral intraventricular':ab,ti OR 'intraventricular hemorrhage, cerebral':ab,ti OR 'cerebral intraventricular haemorrhage':ab,ti OR 'cerebral intraventricular haemorrhages':ab,ti OR 'haemorrhage, cerebral intraventricular':ab,ti OR 'intraventricular haemorrhage, cerebral':ab,ti OR 'cerebral intraventricular hemorrhage':ab,ti OR 'intraventricular haemorrhages, cerebral':ab,ti | 71 |
| #3 | #1 OR #2 | 202,305 |
| #4 | 'prematurity'/exp | 150,025 |
| #5 | 'infant, premature':ab,ti OR 'infants, premature':ab,ti OR 'premature infant':ab,ti OR 'premature infants':ab,ti OR 'preterm infants':ab,ti OR 'infant, preterm':ab,ti OR 'infants, preterm':ab,ti OR 'preterm infant':ab,ti OR 'neonatal prematurity':ab,ti OR 'prematurity, neonatal':ab,ti | 68,856 |
| #6 | #4 OR #5 | 163,012 |
| #7 | 'predict' OR 'prediction' OR 'predictive' OR 'predicted' OR 'prognosis' OR 'prognostic factor' OR 'evaluation' OR 'evaluation study' OR 'risk assessment' OR 'regression analysis' OR 'logistic model' OR 'statistical model' OR 'algorithm' OR 'multivariate analysis' OR 'predictive value of tests' OR 'area under curve' OR 'receiver operator curve' | 7,811,204 |
| #8 | #3 AND #6 AND #7 | 2,890 |
| #7 | human NOT animal | 27,617,131 |
| #8 | #6 AND #7 | 2,776 |
| **Cochrane Library** (2025-2-8) | | |
| #1 | MeSH descriptor: [Cerebral Intraventricular Hemorrhage] explode all trees | 36 |
| #2 | (Cerebral Intraventricular Hemorrhages):ab,ti,kw OR (Hemorrhage, Cerebral Intraventricular):ab,ti,kw OR (Intraventricular Hemorrhage, Cerebral):ab,ti,kw OR (Cerebral Intraventricular Haemorrhage):ab,ti,kw OR (Cerebral Intraventricular Haemorrhages):ab,ti,kw OR (Haemorrhage, Cerebral Intraventricular):ab,ti,kw OR (Intraventricular Haemorrhage, Cerebral):ab,ti,kw OR (Intraventricular Haemorrhages, Cerebral):ab,ti,kw | 644 |
| #3 | #1 or #2 | 644 |
| #4 | (((infant OR infants OR infantile OR infancy OR newb OR "new born" OR "new borns" OR "newly born" OR neonat* OR baby* OR babies OR premature OR prematures OR prematurity OR preterm OR preterms OR "pre term" OR premies OR "low birth weight" OR "low birthweight" OR VLBW OR LBW OR ELBW))):ti,ab | 83,604 |
| #5 | ((("predict" OR "prediction" OR "predictive" OR "predicted" OR "prognosis" OR "prognostic factor" OR "evaluation" OR " evaluation study" OR "risk assessment" OR "regression analysis" OR "logistic model" OR "statistical model" OR "algorithm" OR "multivariate analysis" OR "predictive value of tests" OR "Area Under Curve" OR "Receiver Operator Curve"))) | 460,149 |
| #6 | #3 and #4 and #5 | 105 |
| **Web of science** (2025-2-8) | | |
| #1 | ((TI=(Cerebral Intraventricular Hemorrhages OR Hemorrhage, Cerebral Intraventricular OR Intraventricular Hemorrhage, Cerebral OR Cerebral Intraventricular Haemorrhage OR Cerebral Intraventricular Haemorrhages OR Haemorrhage, Cerebral Intraventricular OR Intraventricular Haemorrhage, Cerebral OR Intraventricular Haemorrhages, Cerebral OR Cerebral Intraventricular Hemorrhage )) OR AB=(Cerebral Intraventricular Hemorrhages OR Hemorrhage, Cerebral Intraventricular OR Intraventricular Hemorrhage, Cerebral OR Cerebral Intraventricular Haemorrhage OR Cerebral Intraventricular Haemorrhages OR Haemorrhage, Cerebral Intraventricular OR Intraventricular Haemorrhage, Cerebral OR Intraventricular Haemorrhages, Cerebral OR Cerebral Intraventricular Hemorrhage )) | 2,847 |
| #2 | ((TI=(prematurity OR Infant, Premature OR Infants, Premature OR Premature Infant OR Premature Infants OR Preterm Infants OR Infant, Preterm OR Infants, Preterm OR Preterm Infant OR Neonatal Prematurity OR Prematurity, Neonatal)) OR AB=(prematurity OR Infant, Premature OR Infants, Premature OR Premature Infant OR Premature Infants OR Preterm Infants OR Infant, Preterm OR Infants, Preterm OR Preterm Infant OR Neonatal Prematurity OR Prematurity, Neonatal)) | 132,025 |
| #3 | ((TI=(predict OR prediction OR predictive OR predicted OR prognosis OR prognostic factor OR evaluation OR evaluation study OR risk assessment OR regression analysis OR logistic model OR statistical model OR algorithm OR multivariate analysis OR predictive value of tests OR area under curve OR receiver operator curve )) OR AB=(predict OR prediction OR predictive OR predicted OR prognosis OR prognostic factor OR evaluation OR evaluation study OR risk assessment OR regression analysis OR logistic model OR statistical model OR algorithm OR multivariate analysis OR predictive value of tests OR area under curve OR receiver operator curve )) | 17,655,173 |
| #4 | #1 and #2 and #3 | 308 |

Chinese Medical Association website using the terms 'intraventricular hemorrhage AND prediction model'.

Cross checking using the terms 'regression analysis and prediction and intraventricular hemorrhage'.
