# Supplementary material for: Prediction models for intraventricular hemorrhage in very preterm infants: a systematic review
Source: Front Pediatr. 2025 Jun 4;13:1605145. doi: 10.3389/fped.2025.1605145 (PMC12174386; doi:10.3389/fped.2025.1605145)
Supplement: Supplementary file 2 [file Table2.docx]

**Appendix Table 2 Studies excluded and reasons for exclusion (Conventional databases)**

| **Study** | **Reasons for Exclusion** |
| --- | --- |
| (Kim et al. 2024) | It is not consistent with the results (intraventricular hemorrhage/death or intraventricular hemorrhage/others) of this study. |
| (Cardoso et al. 2023) |  |
| (Chawla et al. 2022) |  |
| (Iams & Mercer 2003) |  |
| (Bahado-Singh et al. 1998) |  |
| (Yoon et al. 1995) |  |
| (Deng et al. 2024) | The gestational age was not known, and the author was contacted and did not receive a response. |
| (Chen et al. 2025) | The study population was different, with gestational age greater than or equal to 32 weeks. |
| (Metallinou et al. 2024) |  |
| (Puerta-Martínez et al. 2024) |  |
| (Saeedi et al. 2024) |  |
| (Shu et al. 2024) |  |
| (Yang et al. 2024) |  |
| (Arkin et al. 2023) |  |
| (Kumar & Polavarapu 2023) |  |
| (Jiang et al. 2023) |  |
| (Farag et al. 2022) |  |
| (Weinstein et al. 2022) |  |
| (Coskun et al. 2018) |  |
| (Lee et al. 2018) |  |
| (Luque et al. 2014) |  |
| (Singh et al. 2013) |  |
| (Zhang et al. 2013) |  |
| (Bhandari et al. 2011) |  |
| (Chien et al. 2002) |  |
| (Lazzara et al. 1980) |  |
| (Qian et al. 2024) | Not modeled in the study. |
| (Park et al. 2024) |  |
| (Shiono et al. 2024) |  |
| (Suenaga et al. 2024) |  |
| (Alshafei et al. 2023) |  |
| (Depala et al. 2023) |  |
| (Inoue et al. 2023) |  |
| (Tadasa et al. 2023) |  |
| (Zanelli et al. 2023) |  |
| (Shah et al. 2022) |  |
| (Xing et al. 2022) |  |
| (Zhao et al. 2022) |  |
| (Al-Mouqdad et al. 2021) |  |
| (Thornburg et al. 2021) |  |
| (Wolf et al. 2021) |  |
| (Alotaibi et al. 2020) |  |
| (Beltempo et al. 2019) |  |
| (Sloane et al. 2019) |  |
| (Zhi 2019) |  |
| (Boghossian et al. 2018) |  |
| (Morsing et al. 2018) |  |
| (Kim et al. 2018) |  |
| (Poryo et al. 2018) |  |
| (Roberts et al. 2018) |  |
| (Elfarargy et al. 2017) |  |
| (Hannaford et al. 2016) |  |
| (Rhee et al. 2016) |  |
| (Waitz et al. 2016) |  |
| (Ahn et al. 2015) |  |
| (Sarkar et al. 2009) |  |
| (Anteby et al. 2001) |  |
| (Ashoori et al. 2023) | Inconsistent with the diagnostic grading criteria of this study. |
| (Turova et al. 2020) |  |
| (O'Leary et al. 2009) |  |
| (Al-Mouqdad et al. 2024) | Inconsistency in research themes. |
| (Ducatez et al. 2024) |  |
| (Tanaka et al. 2024) |  |
| (Abraham et al. 2023) |  |
| (Akın et al. 2023) |  |
| (Tombolini et al. 2022) |  |
| (Wolf et al. 2021) |  |
| (Sheikhtaheri et al. 2021) |  |
| (Lampe et al. 2020) |  |
| (Turai et al. 2019) |  |
| (Jobe & Goldenberg 2018) |  |
| (Lago et al. 1999) |  |

**Studies excluded and reasons for exclusion (Targeted full-text screening for regression analysis)**

| ***Study*** | ***Reasons for Exclusion*** |
| --- | --- |
| ***(Cucerea et al. 2024)*** | **Repeated with the currently included literature** |
| ***(Heuchan et al. 2002)*** |  |
| ***(Siddappa et al. 2021)*** |  |
| ***(Sidorenko et al. 2024)*** |  |
| *(Hamilton et al. 2020)* | Inconsistency in research themes |
| *(Chien et al. 2002)* | Incorrect population |
| *(Coskun et al. 2018)* |  |
| *(Lee et al. 2018)* |  |
| *(Luque et al. 2014)* |  |
| *(Zernikow et al. 1998)* |  |
| *(Depala et al. 2023)* | Not modeled in the study |
| *(Kovalova et al. 2018)* |  |
| *(Prasun et al. 2018)* |  |

**Abraham BM, Zaazoue MA, Xu G, and Ducis KA**. 2023. Intraventricular hemorrhage in term infants: a single institutional experience between 2016 and 2020. *Childs Nerv Syst* **39**:2123-2129. <http://dx.doi.org/10.1007/s00381-023-05939-8>

**Ahn SY, Shim SY, and Sung IK**. 2015. Intraventricular Hemorrhage and Post Hemorrhagic Hydrocephalus among Very-Low-Birth-Weight Infants in Korea. *J Korean Med Sci* **30 Suppl 1**:S52-58. <http://dx.doi.org/10.3346/jkms.2015.30.S1.S52>

**Akın M, Sarı FN, Ceran B, Bozkaya D, Okman E, Alkan M, and Dizdar EA**. 2023. Cerebral monitoring of very preterm infants with anterior cerebral artery resistive index and early NIRS. *Turk J Med Sci* **53**:225-232. <http://dx.doi.org/10.55730/1300-0144.5577>

**Al-Mouqdad MM, Abdelrahim A, Abdalgader AT, Alyaseen N, Khalil TM, Taha MY, and Asfour SS**. 2021. Risk factors for intraventricular hemorrhage in premature infants in the central region of Saudi Arabia. *Int J Pediatr Adolesc Med* **8**:76-81. <http://dx.doi.org/10.1016/j.ijpam.2019.11.005>

**Al-Mouqdad MM, Alshaikh B, Sumaily HH, Almotiri AA, Alodhaidan NA, AlMahmoud L, Abdelrahim A, Yousif TE, Alghamdi AS, Albarrak YA, Alnafiey AO, Al-Anazi MR, Khalil TM, Asfour RS, and Asfour SS**. 2024. Impact of Refeeding Syndrome on the Short-Term Clinical Outcomes of Very-Premature Infants. *Nutrients* **16**. <http://dx.doi.org/10.3390/nu16203445>

**Alotaibi WSM, Alsaif NS, Ahmed IA, Mahmoud AF, Ali K, Hammad A, Aldibasi OS, and Alsaif SA**. 2020. Reduction of severe intraventricular hemorrhage, a tertiary single-center experience: incidence trends, associated risk factors, and hospital policy. *Childs Nerv Syst* **36**:2971-2979. <http://dx.doi.org/10.1007/s00381-020-04621-7>

**Alshafei A, Farouk S, Khan A, Ahmed M, Elsaba Y, and Aldoky Y**. 2023. Association of umbilical venous catheters vs peripherally inserted central catheters with death or severe intraventricular hemorrhage among preterm infants < 30 weeks: A randomized clinical trial. *J Neonatal Perinatal Med* **16**:247-255. <http://dx.doi.org/10.3233/npm-221126>

**Anteby, II, Anteby EY, Chen B, Hamvas A, McAlister W, and Tychsen L**. 2001. Retinal and intraventricular cerebral hemorrhages in the preterm infant born at or before 30 weeks' gestation. *J aapos* **5**:90-94. <http://dx.doi.org/10.1067/mpa.2001.113841>

**Arkin N, Wang Y, and Wang L**. 2023. Establishment and evaluation of nomogram for predicting intraventricular hemorrhage in neonatal acute respiratory distress syndrome. *BMC Pediatr* **23**:47. <http://dx.doi.org/10.1186/s12887-023-03853-1>

**Ashoori M, O'Toole JM, O'Halloran KD, Naulaers G, Thewissen L, Miletin J, Cheung PY, El-Khuffash A, Van Laere D, Straňák Z, Dempsey EM, and McDonald FB**. 2023. Machine Learning Detects Intraventricular Haemorrhage in Extremely Preterm Infants. *Children (Basel)* **10**. <http://dx.doi.org/10.3390/children10060917>

**Bahado-Singh RO, Dashe J, Deren O, Daftary G, Copel JA, and Ehrenkranz RA**. 1998. Prenatal prediction of neonatal outcome in the extremely low-birth-weight infant. *Am J Obstet Gynecol* **178**:462-468. <http://dx.doi.org/10.1016/s0002-9378(98)70421-1>

**Beltempo M, Wintermark P, Lemyre B, Shalish W, Martel-Bucci A, Narvey M, Ng EH, Guillot M, and Shah PS**. 2019. Predictors of Severe Neurologic Injury on Ultrasound Scan of the Head and Risk Factor-based Screening for Infants Born Preterm. *J Pediatr* **214**:27-33.e23. <http://dx.doi.org/10.1016/j.jpeds.2019.06.065>

**Bhandari V, Buhimschi CS, Han CS, Lee SY, Pettker CM, Campbell KH, Dulay AT, Oliver EA, Werner EF, and Buhimschi IA**. 2011. Cord blood erythropoietin and interleukin-6 for prediction of intraventricular hemorrhage in the preterm neonate. *J Matern Fetal Neonatal Med* **24**:673-679. <http://dx.doi.org/10.3109/14767058.2010.520048>

**Boghossian NS, Geraci M, Edwards EM, and Horbar JD**. 2018. Neonatal and fetal growth charts to identify preterm infants <30 weeks gestation at risk of adverse outcomes. *Am J Obstet Gynecol* **219**:195.e191-195.e114. <http://dx.doi.org/10.1016/j.ajog.2018.05.002>

**Cardoso VC, Grandi C, Silveira RC, Duarte JLB, Viana M, Ferreira D, Alves JMSJ, Embrizi LF, Gimenes CB, de Mello ESNM, Melo FPG, Venzon PS, Gomez DB, Vale MSD, Bentlin MR, Barros MCM, Cardoso L, Diniz EMA, Luz JH, Marba STM, Almeida J, Aragon DC, and Carmona F**. 2023. Growth phenotypes of very low birth weight infants for prediction of neonatal outcomes from a Brazilian cohort: comparison with INTERGROWTH. *J Pediatr (Rio J)* **99**:86-93. <http://dx.doi.org/10.1016/j.jped.2022.07.007>

**Chawla S, Natarajan G, Laptook AR, Chowdhury D, Bell EF, Ambalavanan N, Carlo WA, Gantz M, Das A, Tapia JL, Harmon HM, and Shankaran S**. 2022. Model for severe intracranial hemorrhage and role of early indomethacin in extreme preterm infants. *Pediatr Res* **92**:1648-1656. <http://dx.doi.org/10.1038/s41390-022-02012-z>

**Chen YT, Wu HP, Lan HY, Peng HF, Chen SJ, Yin T, Liaw JJ, and Chang YC**. 2025. Effects of intubation and hypoxemia on intraventricular hemorrhage in preterm infants during the first week: An observational study. *Heart Lung* **69**:78-86. <http://dx.doi.org/10.1016/j.hrtlng.2024.09.013>

**Chien LY, Whyte R, Thiessen P, Walker R, Brabyn D, and Lee SK**. 2002. Snap-II predicts severe intraventricular hemorrhage and chronic lung disease in the neonatal intensive care unit. *J Perinatol* **22**:26-30. <http://dx.doi.org/10.1038/sj.jp.7210585>

**Coskun Y, Isik S, Bayram T, Urgun K, Sakarya S, and Akman I**. 2018. A clinical scoring system to predict the development of intraventricular hemorrhage (IVH) in premature infants. *Childs Nerv Syst* **34**:129-136. <http://dx.doi.org/10.1007/s00381-017-3610-z>

**Cucerea M, Moscalu M, Simon M, Ognean ML, Mitranovici MI, Chiorean DM, and Marian R**. 2024. The Early Hematological Profile and Its Variations: A Useful Tool in the Prediction of Intraventricular Hemorrhage in Extremely Preterm Infants. *Medicina (Kaunas)* **60**. <http://dx.doi.org/10.3390/medicina60030410>

**Deng Z, Tang J, Fang C, and Zhang BH**. 2024. Development and validation of a diagnostic prediction model for severe periventricular-intraventricular hemorrhage in newborns: insights from a retrospective analysis utilizing the MIMIC-III database. *J Pediatr (Rio J)* **100**:327-334. <http://dx.doi.org/10.1016/j.jped.2023.12.004>

**Depala KS, Chintala S, Joshi S, Budhani S, Paidipelly N, Patel B, Rastogi A, Madas N, Vejju R, and Mydam J**. 2023. Clinical Variables Associated With Grade III and IV Intraventricular Hemorrhage (IVH) in Preterm Infants Weighing Less Than 750 Grams. *Cureus* **15**:e40471. <http://dx.doi.org/10.7759/cureus.40471>

**Ducatez F, Tebani A, Abily-Donval L, Snanoudj S, Pilon C, Plichet T, Le Chatelier C, Bekri S, and Marret S**. 2024. New insights and potential biomarkers for intraventricular hemorrhage in extremely premature infant, case-control study. *Pediatr Res* **96**:395-401. <http://dx.doi.org/10.1038/s41390-024-03111-9>

**Elfarargy MS, Eltomey MA, and Soliman NA**. 2017. Early predictors of neonatal intraventricular hemorrhage. *Electron Physician* **9**:4946-4951. <http://dx.doi.org/10.19082/4946>

**Farag MM, Gouda MH, Almohsen AMA, and Khalifa MA**. 2022. Intraventricular hemorrhage prediction in premature neonates in the era of hemodynamics monitoring: a prospective cohort study. *Eur J Pediatr* **181**:4067-4077. <http://dx.doi.org/10.1007/s00431-022-04630-5>

**Hamilton EF, Dyachenko A, Ciampi A, Maurel K, Warrick PA, and Garite TJ**. 2020. Estimating risk of severe neonatal morbidity in preterm births under 32 weeks of gestation. *J Matern Fetal Neonatal Med* **33**:73-80. <http://dx.doi.org/10.1080/14767058.2018.1487395>

**Hannaford KE, Stout MJ, Smyser CD, Mathur A, and Cahill AG**. 2016. Evaluating the Sensitivity of Electronic Fetal Monitoring Patterns for the Prediction of Intraventricular Hemorrhage. *Am J Perinatol* **33**:1420-1425. <http://dx.doi.org/10.1055/s-0036-1584140>

**Heuchan AM, Evans N, Henderson Smart DJ, and Simpson JM**. 2002. Perinatal risk factors for major intraventricular haemorrhage in the Australian and New Zealand Neonatal Network, 1995-97. *Arch Dis Child Fetal Neonatal Ed* **86**:F86-90. <http://dx.doi.org/10.1136/fn.86.2.f86>

**Iams JD, and Mercer BM**. 2003. What we have learned about antenatal prediction of neonatal morbidity and mortality. *Semin Perinatol* **27**:247-252. <http://dx.doi.org/10.1016/s0146-0005(03)00019-3>

**Inoue T, Nishikubo T, Hirano S, Kamamoto T, Takahashi Y, and Kusuda S**. 2023. Risk factor analyses for intraventricular hemorrhage in preterm infants: A retrospective cohort study. *Pediatr Int* **65**:e15599. <http://dx.doi.org/10.1111/ped.15599>

**Jiang L, Yu Q, Wang F, Wu M, Liu F, Fu M, Gao J, Feng X, Zhang L, and Xu Z**. 2023. The role of blood pressure variability indicators combined with cerebral blood flow parameters in predicting intraventricular hemorrhage in very low birth weight preterm infants. *Front Pediatr* **11**:1241809. <http://dx.doi.org/10.3389/fped.2023.1241809>

**Jobe AH, and Goldenberg RL**. 2018. Antenatal corticosteroids: an assessment of anticipated benefits and potential risks. *Am J Obstet Gynecol* **219**:62-74. <http://dx.doi.org/10.1016/j.ajog.2018.04.007>

**Kim HH, Kim JK, and Park SY**. 2024. Predicting severe intraventricular hemorrhage or early death using machine learning algorithms in VLBWI of the Korean Neonatal Network Database. *Sci Rep* **14**:11113. <http://dx.doi.org/10.1038/s41598-024-62033-y>

**Kim SM, Sung JH, Kuk JY, Cha HH, Choi SJ, Oh SY, and Roh CR**. 2018. Short- and long-term neonatal outcomes according to differential exposure to antenatal corticosteroid therapy in preterm births prior to 24 weeks of gestation. *PLoS One* **13**:e0198471. <http://dx.doi.org/10.1371/journal.pone.0198471>

**Kovalova OM, Pokhylko VI, Bielorus AI, Soloviova HO, Cherniavska YI, and Adamchuk NN**. 2018. [Predicting the occurrence of severe intraventricular hemorrhages and ways to prevent their development in preterm infants]. *Wiad Lek* **71**:1524-1530.

**Kumar P, and Polavarapu M**. 2023. A simple scoring system for prediction of IVH in very-low-birth-weight infants. *Pediatr Res* **94**:2033-2039. <http://dx.doi.org/10.1038/s41390-023-02744-6>

**Lago P, Freato F, Bettiol T, Chiandetti L, Vianello A, and Zaramella P**. 1999. Is the CRIB score (clinical risk index for babies) a valid tool in predicting neurodevelopmental outcome inExtremely low birth weight infants? *Biol Neonate* **76**:220-227. <http://dx.doi.org/10.1159/000014162>

**Lampe R, Rieger-Fackeldey E, Sidorenko I, Turova V, Botkin N, Eckardt L, Alves-Pinto A, Kovtanyuk A, Schündeln M, and Felderhoff-Müser U**. 2020. Assessing key clinical parameters before and after intraventricular hemorrhage in very preterm infants. *Eur J Pediatr* **179**:929-937. <http://dx.doi.org/10.1007/s00431-020-03585-9>

**Lazzara A, Ahmann P, Dykes F, Brann AW, Jr., and Schwartz J**. 1980. Clinical predictability of intraventricular hemorrhage in preterm infants. *Pediatrics* **65**:30-34.

**Lee J, Hong M, Yum SK, and Lee JH**. 2018. Perinatal prediction model for severe intraventricular hemorrhage and the effect of early postnatal acidosis. *Childs Nerv Syst* **34**:2215-2222. <http://dx.doi.org/10.1007/s00381-018-3868-9>

**Luque MJ, Tapia JL, Villarroel L, Marshall G, Musante G, Carlo W, and Kattan J**. 2014. A risk prediction model for severe intraventricular hemorrhage in very low birth weight infants and the effect of prophylactic indomethacin. *J Perinatol* **34**:43-48. <http://dx.doi.org/10.1038/jp.2013.127>

**Metallinou D, Karampas G, Pavlou ML, Louma MI, Mantzou A, Sarantaki A, Nanou C, Gourounti K, Tzeli M, Pantelaki N, Tzamakos E, Boutsikou T, Lykeridou A, and Iacovidou N**. 2024. Serum Neuron-Specific Enolase as a Biomarker of Neonatal Brain Injury-New Perspectives for the Identification of Preterm Neonates at High Risk for Severe Intraventricular Hemorrhage. *Biomolecules* **14**. <http://dx.doi.org/10.3390/biom14040434>

**Morsing E, Maršál K, and Ley D**. 2018. Reduced Prevalence of Severe Intraventricular Hemorrhage in Very Preterm Infants Delivered after Maternal Preeclampsia. *Neonatology* **114**:205-211. <http://dx.doi.org/10.1159/000489039>

**O'Leary H, Gregas MC, Limperopoulos C, Zaretskaya I, Bassan H, Soul JS, Di Salvo DN, and du Plessis AJ**. 2009. Elevated cerebral pressure passivity is associated with prematurity-related intracranial hemorrhage. *Pediatrics* **124**:302-309. <http://dx.doi.org/10.1542/peds.2008-2004>

**Park J, Park SH, Kwon YR, Yoon SJ, Lim JH, Han JH, Shin JE, Eun HS, Park MS, and Lee SM**. 2024. Long-term outcomes of very low birth weight infants with intraventricular hemorrhage: a nationwide population study from 2011 to 2019. *World J Pediatr* **20**:692-700. <http://dx.doi.org/10.1007/s12519-024-00799-x>

**Poryo M, Boeckh JC, Gortner L, Zemlin M, Duppré P, Ebrahimi-Fakhari D, Wagenpfeil S, Heckmann M, Mildenberger E, Hilgendorff A, Flemmer AW, Frey G, and Meyer S**. 2018. Ante-, peri- and postnatal factors associated with intraventricular hemorrhage in very premature infants. *Early Hum Dev* **116**:1-8. <http://dx.doi.org/10.1016/j.earlhumdev.2017.08.010>

**Prasun P, Madan R, Puthuraya S, Subramanian D, Datta I, Kalra V, Thomas R, Stockton DW, Sundaram S, Callaghan J, Callaghan M, and Chouthai N**. 2018. Can Functional Polymorphisms in VEGF and MMP Predict Intraventricular Hemorrhage in Extremely Preterm Newborns? *Dev Neurosci* **40**:337-343. <http://dx.doi.org/10.1159/000493788>

**Puerta-Martínez AG, López-Garrido E, Guerrero-Nava JM, Vargas-Ruiz R, and Martínez-Padrón HY**. 2024. Risk factors associated with intraventricular hemorrhage in very-low-birth-weight premature infants. *Childs Nerv Syst* **40**:1743-1750. <http://dx.doi.org/10.1007/s00381-024-06310-1>

**Qian Y, Huang J, Cheng H, and Wang J**. 2024. Effect of days of age at first blood transfusion on intraventricular hemorrhage in very low and extremely low birth weight infants. *Expert Rev Hematol* **17**:871-875. <http://dx.doi.org/10.1080/17474086.2024.2422017>

**Rhee CJ, Kibler KK, Easley RB, Andropoulos DB, Czosnyka M, Smielewski P, Varsos GV, Brady KM, Rusin CG, Fraser CD, 3rd, Gauss CH, Williams DK, and Kaiser JR**. 2016. The Diastolic Closing Margin Is Associated with Intraventricular Hemorrhage in Premature Infants. *Acta Neurochir Suppl* **122**:147-150. <http://dx.doi.org/10.1007/978-3-319-22533-3_30>

**Roberts JC, Javed MJ, Hocker JR, Wang H, and Tarantino MD**. 2018. Risk factors associated with intraventricular hemorrhage in extremely premature neonates. *Blood Coagul Fibrinolysis* **29**:25-29. <http://dx.doi.org/10.1097/mbc.0000000000000661>

**Saeedi E, Mashhadinejad M, and Tavallaii A**. 2024. Development of a machine learning model for prediction of intraventricular hemorrhage in premature neonates. *Childs Nerv Syst* **41**:51. <http://dx.doi.org/10.1007/s00381-024-06714-z>

**Sarkar S, Bhagat I, Dechert R, Schumacher RE, and Donn SM**. 2009. Severe intraventricular hemorrhage in preterm infants: comparison of risk factors and short-term neonatal morbidities between grade 3 and grade 4 intraventricular hemorrhage. *Am J Perinatol* **26**:419-424. <http://dx.doi.org/10.1055/s-0029-1214237>

**Shah V, Musrap N, Maharaj K, Afifi J, El-Naggar W, Kelly E, Mukerji A, Shah P, and Vincer M**. 2022. Grading of Intraventricular Hemorrhage and Neurodevelopment in Preterm <29 Weeks’ GA in Canada. *Children (Basel)* **9**. <http://dx.doi.org/10.3390/children9121948>

**Sheikhtaheri A, Zarkesh MR, Moradi R, and Kermani F**. 2021. Prediction of neonatal deaths in NICUs: development and validation of machine learning models. *BMC Med Inform Decis Mak* **21**:131. <http://dx.doi.org/10.1186/s12911-021-01497-8>

**Shiono A, Bonno M, Toyoda H, Ogawa M, Tanaka S, and Hirayama M**. 2024. Autonomic Nervous System in Preterm Very Low Birth Weight Neonates with Intraventricular Hemorrhage. *Am J Perinatol* **41**:e577-e583. <http://dx.doi.org/10.1055/a-1926-0335>

**Shu CH, Zebda R, Espinosa C, Reiss J, Debuyserie A, Reber K, Aghaeepour N, and Pammi M**. 2024. Early prediction of mortality and morbidities in VLBW preterm neonates using machine learning. *Pediatr Res*. <http://dx.doi.org/10.1038/s41390-024-03604-7>

**Siddappa AM, Quiggle GM, Lock E, and Rao RB**. 2021. Predictors of severe intraventricular hemorrhage in preterm infants under 29-weeks gestation. *J Matern Fetal Neonatal Med* **34**:195-200. <http://dx.doi.org/10.1080/14767058.2019.1601698>

**Sidorenko I, Brodkorb S, Felderhoff-Müser U, Rieger-Fackeldey E, Krüger M, Feddahi N, Kovtanyuk A, Lück E, and Lampe R**. 2024. Assessment of intraventricular hemorrhage risk in preterm infants using mathematically simulated cerebral blood flow. *Front Neurol* **15**:1465440. <http://dx.doi.org/10.3389/fneur.2024.1465440>

**Singh R, Gorstein SV, Bednarek F, Chou JH, McGowan EC, and Visintainer PF**. 2013. A predictive model for SIVH risk in preterm infants and targeted indomethacin therapy for prevention. *Sci Rep* **3**:2539. <http://dx.doi.org/10.1038/srep02539>

**Sloane AJ, Flannery DD, Lafferty M, Jensen EA, Dysart K, Cook A, Greenspan J, and Aghai ZH**. 2019. Hypertensive disorders during pregnancy are associated with reduced severe intraventricular hemorrhage in very-low-birth-weight infants. *J Perinatol* **39**:1125-1130. <http://dx.doi.org/10.1038/s41372-019-0413-y>

**Suenaga H, Nakanishi H, Uchiyama A, and Kusuda S**. 2024. Small for Gestational Age Affects Outcomes on Singletons and Inborn Births in Extremely Preterm Infants: A Japanese Cohort Study. *Am J Perinatol* **41**:e780-e787. <http://dx.doi.org/10.1055/a-1933-4627>

**Tadasa S, Tilahun H, Melkie M, Getachew S, Debele GR, and Bekele F**. 2023. Magnitude and associated factors of intraventricular hemorrhage in preterm neonates admitted to low resource settings: a cross-sectional study. *Ann Med Surg (Lond)* **85**:2534-2539. <http://dx.doi.org/10.1097/ms9.0000000000000774>

**Tanaka K, Matsumoto S, Minamitani Y, Imamura H, Yoshimatsu H, Nakamura T, Naramura T, Iwai M, Mitsubuchi H, and Nakamura K**. 2024. Changes in Internal Cerebral Vein Pulsation and Intraventricular Hemorrhage in Extremely Preterm Infants. *Am J Perinatol* **41**:e37-e45. <http://dx.doi.org/10.1055/a-1817-6638>

**Thornburg CD, Erickson SW, Page GP, Clark EAS, DeAngelis MM, Hartnett ME, Goldstein RF, Dagle JM, Murray JC, Poindexter BB, Das A, and Cotten CM**. 2021. Genetic predictors of severe intraventricular hemorrhage in extremely low-birthweight infants. *J Perinatol* **41**:286-294. <http://dx.doi.org/10.1038/s41372-020-00821-w>

**Tombolini S, De Angelis F, Correani A, Marchionni P, Monachesi C, Ferretti E, Staffolani F, D'Ascenzo R, and Carnielli V**. 2022. Is low cerebral near infrared spectroscopy oximetry associated with neurodevelopment of preterm infants without brain injury? *J Perinat Med* **50**:625-629. <http://dx.doi.org/10.1515/jpm-2021-0498>

**Turai R, Schandl MF, Dergez T, Vass RA, Kvárik T, Horányi E, Balika D, Mammel B, Gyarmati J, Fónai F, Vida G, Funke S, Gaál V, Reglődi D, and Ertl T**. 2019. [Early and late complications of hyperglycemic extremely low birth-weight infants]. *Orv Hetil* **160**:1270-1278. <http://dx.doi.org/10.1556/650.2019.31455>

**Turova V, Sidorenko I, Eckardt L, Rieger-Fackeldey E, Felderhoff-Müser U, Alves-Pinto A, and Lampe R**. 2020. Machine learning models for identifying preterm infants at risk of cerebral hemorrhage. *PLoS One* **15**:e0227419. <http://dx.doi.org/10.1371/journal.pone.0227419>

**Waitz M, Nusser S, Schmid MB, Dreyhaupt J, Reister F, and Hummler H**. 2016. Risk Factors Associated with Intraventricular Hemorrhage in Preterm Infants with ≤28 Weeks Gestational Age. *Klin Padiatr* **228**:245-250. <http://dx.doi.org/10.1055/s-0042-111689>

**Weinstein RM, Parkinson C, Everett AD, Graham EM, Vaidya D, and Northington FJ**. 2022. A predictive clinical model for moderate to severe intraventricular hemorrhage in very low birth weight infants. *J Perinatol* **42**:1374-1379. <http://dx.doi.org/10.1038/s41372-022-01435-0>

**Wolf HT, Weber T, Schmidt S, Norman M, Varendi H, Piedvache A, Zeitlin J, and Huusom LD**. 2021. Mode of delivery and adverse short- and long-term outcomes in vertex-presenting very preterm born infants: a European population-based prospective cohort study. *J Perinat Med* **49**:923-931. <http://dx.doi.org/10.1515/jpm-2020-0468>

**Xing S, Sun HQ, and Li MC**. 2022. [Clinical characteristics and risk factors of periventricular-intraventricular hemorrhage in extremely low birth weight infants]. *Zhonghua Yi Xue Za Zhi* **102**:3774-3778. <http://dx.doi.org/10.3760/cma.j.cn112137-20220616-01323>

**Yang YH, Wang TT, Su YH, Chu WY, Lin WT, Chen YJ, Chang YS, Lin YC, Lin CH, and Lin YJ**. 2024. Predicting early mortality and severe intraventricular hemorrhage in very-low birth weight preterm infants: a nationwide, multicenter study using machine learning. *Sci Rep* **14**:10833. <http://dx.doi.org/10.1038/s41598-024-61749-1>

**Yoon BH, Romero R, Kim CJ, Jun JK, Gomez R, Choi JH, and Syn HC**. 1995. Amniotic fluid interleukin-6: A sensitive test for antenatal diagnosis of acute inflammatory lesions of preterm placenta and prediction of perinatal morbidity. *American Journal of Obstetrics and Gynecology* **172**:960-970. <http://dx.doi.org/10.1016/0002-9378(95)90028-4>

**Zanelli SA, Abubakar M, Andris R, Patwardhan K, Fairchild KD, and Vesoulis ZA**. 2023. Early Vital Sign Differences in Very Low Birth Weight Infants with Severe Intraventricular Hemorrhage. *Am J Perinatol* **40**:1193-1201. <http://dx.doi.org/10.1055/s-0041-1733955>

**Zernikow B, Holtmannspoetter K, Michel E, Theilhaber M, Pielemeier W, and Hennecke KH**. 1998. Artificial neural network for predicting intracranial haemorrhage in preterm neonates. *Acta Paediatr* **87**:969-975. <http://dx.doi.org/10.1080/080352598750031644>

**Zhang Y, Chen M, Hu Q, and Huang W**. 2013. Detection and quantification of intracerebral and intraventricular hemorrhage from computed tomography images with adaptive thresholding and case-based reasoning. *Int J Comput Assist Radiol Surg* **8**:917-927. <http://dx.doi.org/10.1007/s11548-013-0830-x>

**Zhao Y, Zhang W, and Tian X**. 2022. Analysis of risk factors of early intraventricular hemorrhage in very-low-birth-weight premature infants: a single center retrospective study. *BMC Pregnancy Childbirth* **22**:890. <http://dx.doi.org/10.1186/s12884-022-05245-2>

**Zhi ZEKZ**. 2019. [Incidence and risk factors of severe intraventricular hemorrhage in very low and extremely low birth weight infants: a multi-center study]. *Zhonghua Er Ke Za Zhi* **57**:258-264. <http://dx.doi.org/10.3760/cma.j.issn.0578-1310.2019.04.006>
