# Supplementary material for: Prediction models for intraventricular hemorrhage in very preterm infants: a systematic review
Source: Front Pediatr. 2025 Jun 4;13:1605145. doi: 10.3389/fped.2025.1605145 (PMC12174386; doi:10.3389/fped.2025.1605145)
Supplement: Supplementary file 4 [file Supplementaryfile4.docx]

**Appendix Table 3 Predictors and number of predictors in the original study**

| **Study** | **Predictors and number of candidates** | **Predictors and numbers of the model** |
| --- | --- | --- |
| Cucerea et al. | Gestational age [GA], Mechanical ventilation [MV], hypercapnia, hypotension, potential of hydrogen [Ph], Base Excess [BE], cord lactate, initial hematocrit [HCT], hemoglobin [HGB], erythrocytes. (10) | GA, hypercapnia, hypotension, BE, HCT. (5) |
| Han et al. | Birth weight [BW], Sex, Gestational age [GA],Apgar score at 1 minute, Apgar score at 5 minutes, Maternal age, In vitro fertilization, Premature rupture of membrane, Pregnancy-induced hypertension, Gestational diabetes mellitus, Histological chorioamnionitis, Administration of antenatal steroids, Resuscitation in the delivery room, Surfactant use, Inotropes, Antibiotics, Sedatives, Neuromuscular blockers, Systemic steroids, Ventilator modes, Mean airway pressure [MAP], Fraction of inspired oxygen [FiO₂], pH, Hemoglobin, Potassium, Chloride, Bicarbonate, Systolic blood pressure [SBP], Diastolic blood pressure [DBP], Mean blood pressure [MBP], Heart rate [HR], Respiratory rate [RR], Body temperature [BT], Percutaneous oxygen saturation [SpO₂], Duration of NICU stay. (29) | SBP, DBP, MBP, HR, RR, BT, SpO2, MAP, FiO2, inotropes, antibiotics, sedatives, neuromuscular blockers, systemic steroids. (14) |
|  |  | SBP, DBP, MBP, HR, RR, BT, SpO2, MAP, FiO2, inotropes, antibiotics, sedatives, neuromuscular blockers, systemic steroids. (14) |
|  |  | SBP, DBP, MBP, HR, RR, BT, SpO2, MAP, FiO2, inotropes, antibiotics, sedatives, neuromuscular blockers, systemic steroids. (14) |
|  |  | SBP, DBP, MBP, HR, RR, BT, SpO2, MAP, FiO2, inotropes, antibiotics, sedatives, neuromuscular blockers, systemic steroids. (14) |
|  |  | SBP, DBP, MBP, HR, RR, BT, SpO2, MAP, FiO2, inotropes, antibiotics, sedatives, neuromuscular blockers, systemic steroids. (14) |
| Sidorenko et al. | Apgar at 1 min, Apgar at 5 min, Apgar at 10 min, focal/spontaneous intestinal perforation [FIP/SIP], cholestasis, acidosis, sex, pulmonary hemorrhage, potential of hydrogen [PH], Leukocytes, Partial Pressure of Carbon Dioxide [PCO2], Partial Pressure of Oxygen [PO2], Thrombocytes, Hematocrit, Cerebral blood flow [CBF], Mean airway pressure [MAP], C-reactive protein [CRP]. (17) | MAP, PH, CRP, CBF, Leukocytes. (5) |
|  |  | Apgar at 10 min, FIP/SIP, cholestasis, acidosis, sex, pulmonary hemorrhage. (6) |
|  |  | Apgar at 10 min, pH, FIP/SIP, Leukocytes. (4) |
|  |  | Apgar at 10 min, pH, Leukocytes. (3) |
|  |  | MAP, pH, CRP, FIP/SIP,  sex, Leukocytes. (6) |
|  |  | MAP, pH, CRP, Leukocytes. (4) |
|  |  | MAP, pH, CRP, CBF,  FIP/SIP, Leukocytes. (6) |
| Wang et al. | sex, gestational complications, multiple pregnancies, mode of delivery, premature rupture of fetal membranes [PROM], amniotic fluid infection, birth asphyxia, administration of pulmonary surfactant，acidosis, mechanical ventilation days [MV], gestational age [GA], birth weight [BW], antenatal corticosteroid. (13) | Mechanical ventilation days, GA, BW, antenatal corticosteroid. (4) |
| Ushida et al. | maternal age, gestational age [GA], parity, delivery mode, gestational diabetes mellitus/ diabetes mellitus, hypertensive disorders of pregnancy [HDP], clinical chorioamnionitis [CAM], premature rupture of membrane [PROM], antenatal corticosteroid treatment [ACS treatment], singleton or twin birth, infant sex ,birth weight [BW]. (12) | maternal age, GA, parity, delivery mode, gestational diabetes mellitus/ diabetes mellitus, HDP, CAM, PROM, ACS treatment, singleton or twin birth, infant sex, BW. (12) |
|  |  | maternal age, GA, parity, delivery mode, gestational diabetes mellitus/ diabetes mellitus, HDP, CAM, PROM, ACS treatment, singleton or twin birth, infant sex, BW. (12) |

**Appendix Table 3 Predictors and number of predictors in the original study (continued)**

| **Study** | **Predictors and number of candidates** | **Predictors and numbers of the model** |
| --- | --- | --- |
|  |  | maternal age, GA, parity, delivery mode, gestational diabetes mellitus/ diabetes mellitus, HDP, CAM, PROM, ACS treatment, singleton or twin birth, infant sex, BW. (12) |
|  |  | maternal age, GA, parity, delivery mode, gestational diabetes mellitus/ diabetes mellitus, HDP, CAM, PROM, ACS treatment, singleton or twin birth, infant sex, BW. (12) |
|  |  | maternal age, GA, parity, delivery mode, gestational diabetes mellitus/ diabetes mellitus, HDP, CAM, PROM, ACS treatment, singleton or twin birth, infant sex, BW. (12) |
|  |  | maternal age, GA, parity, delivery mode, gestational diabetes mellitus/ diabetes mellitus, HDP, CAM, PROM, ACS treatment, singleton or twin birth, infant sex, BW. (12) |
| Liu et al. | gestational age [GA], cesarean delivery, Apgar at 5 min, invasive mechanical ventilation,  Use vasoactive drugs within 24 hours after birth, Erythrocyte hemoglobin concentration, platelet, lactate. (8) | GA, cesarean delivery, Use vasoactive drugs within 24 hours after birth, Erythrocyte hemoglobin concentration, lactate. (5) |
| Ushida et al. | maternal age, gestational age[GA], parity, delivery mode, gestational diabetes mellitus or diabetes mellitus, hypertensive disorders of pregnanc [HDP], clinical chorioamnioniti, premature rupture of membrane [PROM], antenatal corticosteroid treatment , singleton/twins, monochorionic [MC] twins, dichorionic [DC] twins, predicted fetal sex and estimated fetal weight. (12) | GA, HDP, PROM, antenatal corticosteroid treatment, singleton/twins, infant sex. (6) |
| He et al. | antenatal steroid therapy, Gestational age [GA] <28 wk, Birth weight [BW]<1000 g, 1-min Apgar score <8, mechanical ventilation [MV], hypotension, In vitro fertilization, 5-min Apgar <8, Sex [male], Hypoglycemia. (10) | antenatal steroid therapy, GA <28 wk, BW <1000 g, 1-min Apgar score <8, mechanical ventilation and hypotension. (6) |
| Huvanandana et al. | The features evaluated comprised of the mean (μ) as well as short- and long-term scaling exponents derived from detrended fluctuation analysis [DFA] (α1 and α3, respectively), extracted from five different time series.These were: mean arterial pressure [MAP], systolic blood pressure [SBP], diastolic blood pressure [DBP] and pulse interval [PI] series as derived from the arterial blood pressure data, as well as the interbreath intervals [IBI] from the respiratory air flow data. (15) | PI α2, DBP μ. (2) |
|  |  | SBP α1, DBP μ. (2) |
|  |  | PI α1, DBP μ. (2) |
|  |  | DBP α1, DBP μ. (2) |
|  |  | MAP α1, DBP μ. (2) |

**Appendix Table 3 Predictors and number of predictors in the original study (continued)**

| **Study** | **Predictors and number of candidates** | **Predictors and numbers of the model** |
| --- | --- | --- |
| Heuchan et al. | fetal distress, intrauterine growth restriction, antenatal corticosteroids, gestational age [GA], 1 minute Apgar score <4, male sex, prolonged rupture of membranes [PROM], transfer after birth, pregnancy-induced hypertension [PIH], mode of delivery, birth order, 5 minute Apgar score, birth weight [BW], birth weight centile. (14) | GA, antenatal corticosteroids, transfer after birth, 1 minute Apgar score < 4, and sex. (5) |
| Van et al. | Gestational age [GA], birth weight [BW], sex, Apgar score at 5min<7, Idiopathic respiratory distress syndrome [RDS], smoking habits of the mother, preeclampsia, tocolysis with betamimetics or indomethacin, prolonged rupture of membranes [PROM], fetal heart rate pattern abnormalities before labor, fetal heart rate pattern abnormalities during labor, mode of delivery. (12) | GA, BW, sex, Apgar score at 5min<7, RDS. (5) |
